# Red Blood Cell Morphodynamics in Patients with Polycythemia Vera and Stroke

**DOI:** 10.3390/ijms23042247

**Published:** 2022-02-17

**Authors:** Polina I. Kuznetsova, Anton A. Raskurazhev, Alla A. Shabalina, Anait L. Melikhyan, Irina N. Subortseva, Marine M. Tanashyan

**Affiliations:** 1 Research Center of Neurology, 125367 Moscow, Russia; rasckey@live.com (A.A.R.); ashabalina@yandex.ru (A.A.S.); m_tanashyan2004@mail.ru (M.M.T.); 2 National Research Center for Hematology, 125367 Moscow, Russia; anoblood@mail.ru (A.L.M.); soubortseva@yandex.ru (I.N.S.)

**Keywords:** Ph-negative myeloproliferative neoplasms, hematology, brain infarcts, erythrocyte deformability, polycythemia vera, LoRRca

## Abstract

Polycythemia vera (PV) is a Ph-negative myeloproliferative neoplasm (MPN) which is characterized by erythrocytosis and a high incidence of thrombotic complications, including stroke. The study aimed to evaluate red blood cell (RBC) morphodynamic properties in PV patients and their possible association with stroke. We enrolled 48 patients with PV in this cross-sectional study, 13 of which have a history of ischemic stroke. The control group consisted of 90 healthy subjects. RBC deformability and aggregation analysis were performed using a laser-assisted optical rotational red cell analyzer. The following parameters were calculated: aggregation amplitude (Amp), RBC rouleaux formation time constant (Tf), time of formation of three-dimensional aggregates (Ts), aggregation index (AI), rate of complete disaggregation (y-dis), and the maximal elongation of RBC (EImax). Statistical analysis was performed with the R programming language. There were significant differences in RBCs morphodynamics features between patients with PV and the control group. Lower EImax (0.47 (0.44; 0.51) vs. 0.51 (0.47; 0.54), *p* < 0.001) and γ-dis (100 (100; 140) vs. 140 (106; 188) s^−1^, *p* < 0.001) along with higher amplitude (10.1 (8.6; 12.2) vs. 7.7 (6.6; 9.2), *p* < 0.001) was seen in patients with PV compared with control. A statistically significant difference between PV patients with and without stroke in aggregation amplitude was found (*p* = 0.03). A logistic regression model for stroke was built based on RBC morphodynamics which performed reasonably well (*p* = 0.01)**.** RBC alterations may be associated with overt cerebrovascular disease in PV, suggesting a possible link between erythrocyte morphodynamics and increased risk of stroke.

## 1. Introduction

Philadelphia-negative myeloproliferative neoplasms (Ph-negative MPN) are rare clonal hematologic malignancies originating at the pluripotent hematopoietic stem cell level. Classical Ph-negative MPN includes polycythemia vera (PV), essential thrombocythemia (ET), primary (idiopathic) myelofibrosis (PMF), and myeloproliferative neoplasm, unclassified [1,2]. In patients with essential thrombocythemia or primary myelofibrosis, mutations in the Janus kinase 2 (*JAK2*) gene, mutations in exon 9 of the calreticulin gene, and activating mutations in the thrombopoietin receptor gene (MPL) are detected. Patients with polycythemia vera have mutations in exons 12 or 14 of gene *JAK2*. However, there is a proportion of patients without a specific molecular marker identified. In all cases of MPN, there is a dysregulation of the signaling pathway and, as a result, an increase in the transcription of key genes in the cell, which leads to an increase in the level of cytokines, uncontrolled proliferation, and the clinical phenotype of MPN [3]. As a rule, the condition of patients with MPN deteriorates over time, with the accumulation of pathological blood cells in the bone marrow and the peripheral blood [4].

We focused on the so-called Philadelphia chromosome-negative or *BCR-ABL1*-negative MPN—polycythemia vera (PV). The disease is characterized by changes in erythropoiesis, quantitative and qualitative disorders of red blood cells, high hemoglobin levels, and high hematocrit. In patients with PV, the risk of arterial thrombosis was approximately three times higher, respectively, compared with the age-matched and sex-matched control group [5].

The most prevalent complications in patients with PV compared with other MPN are thromboembolic events and cardiovascular disease which can worsen the course of the disease and often cause mortality in this population. Stroke can be the presenting manifestation or may appear as a complication in the course of the hematological disorder [6]. Moreover, a vascular complication, such as arterial or venous thrombosis, often leads to the diagnosis of PV with arterial or venous thrombosis being the most frequent clinical complication [7]. Thrombosis or thromboembolism occurs in almost 41% of patients with PV [8]. Arterial thromboses comprise 60–70% of all cardiovascular events in patients with PV and include transient ischemic attack, stroke, acute myocardial infarction, and peripheral arterial occlusion [9,10,11]. Worldwide incidence and prevalence data are inconclusive. For example, the incidence of PV and ET is 1.0–2.0 per 100,000 person every year in the U.S., while the incidence of PMF is 0.3 per 100,000 person every year [12]. Epidemiological data on cerebrovascular pathology in Ph-negative MPN are sparse and usually deal with symptomatic stroke.

In recent years, a substantial contribution of red blood cells (RBCs) in cardio- and cerebrovascular homeostasis has been evidenced, as these cells can regulate vascular function by the export of nitric oxide. Among the factors comprising RBC morphodynamics, Kim et al. proposed the following: RBC geometry, hemoglobin concentration, rheological properties of the RBC membrane, osmotic concentrations, calcium, nitric oxide (and consequently, endothelial function), temperature, alteration of membrane proteins, lipids, erythrocyte ATP, and erythrocyte aging [13]. RBCs play an important role in vascular events due to their properties to deformity its shape, which determines their ability to perform a transport function. Changing erythrocyte’s shape at a constant volume and surface area allows them to adapt to the conditions of blood flow in the microcirculation system. The deformability of RBC depends on many factors (internal viscosity, cellular geometry, membrane properties, etc.). Abnormal RBC deformability contributes to hemolysis, thrombophilia, inflammation, and microvascular occlusion in various circulatory diseases [14]. In this regard, the deformability of RBC is also a mechanism that may affect thrombotic events [15,16,17]. This new evidence increases interest in searching new triggers or possible predictive markers that may identify high-risk stroke patients with PV.

In this cross-sectional study, we aim to evaluate the deformability of red blood cells and their contribution to the realization of stroke and other thrombotic complications in patients with PV.

## 2. Results

**Figure 1:**
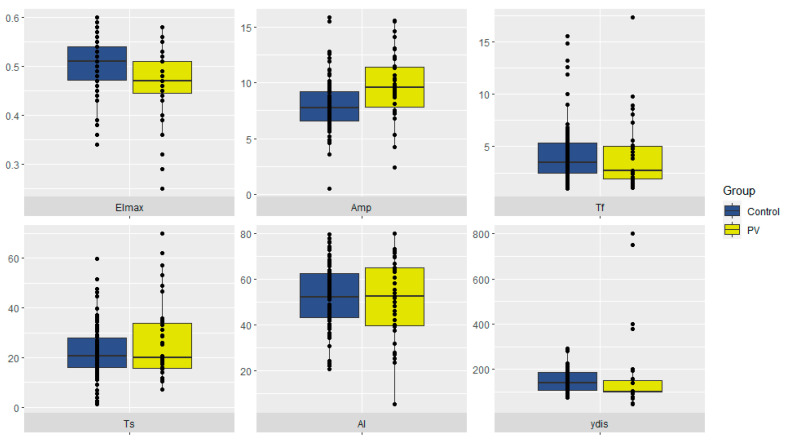
RBC morphodynamics in PV patients and control. RBCs—red blood cells. PV—polycythemia vera.

The initial step in our study was to identify possible differences in RBC morphodynamics between PV patients (*n* = 48) and healthy controls (*n* = 90). The main findings are summarized in Table 1 and Figure 1.

Lower RBC deformability (EImax) and lower rates of complete disaggregation (γ-dis), along with a higher aggregation amplitude (Amp), were seen in patients with PV compared with control (*p* < 0.001).

**Figure 2:**
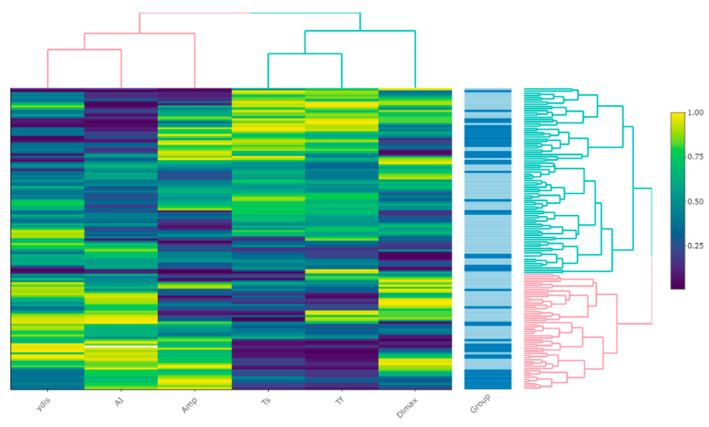
A heatmap of the relative values of RBC morphodynamics parameters expressed as percentiles. The ‘Group’ column represents rows of observations, with darker colors indicating PV patients. The dendrograms represent the process of clustering with color defining major clusters.

Hierarchical clustering (visualized via heatmap [Figure 2]) did not reveal any explicit patterns for PV; however, a tendency towards median values of most parameters (‘green’) may be observed in the middle, corresponding to the control group subjects.

**Figure 3:**
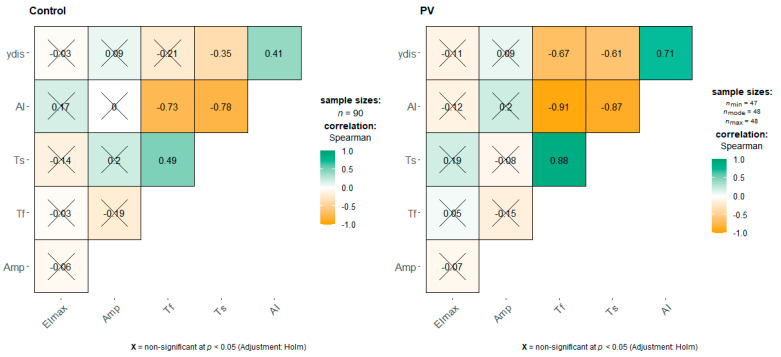
Correlation plot of RBC morphodynamic properties in PV patients and control. Numbers indicate the Spearman rank correlation coefficient, crossed-out are non-significant (at *p* < 0.05) correlations. Green (brown) color indicates positive (negative) correlations, color intensity represents their strength.

We performed a correlation analysis (Figure 3), which showed a more prominent negative correlation between Tf and γ-dis (ρ = −0.67) in the PV group than in the control (ρ = −0.21)—the latter was statistically insignificant. In both groups, there were positive statistically significant correlations between Tf and Ts (ρ = 0.88 vs. ρ = 0.49), and between AI and ydis (ρ = 0.71 vs. ρ = 0.41), but there were stronger correlations in the PV group.

The second step of our study included an analysis of the possible association of RBC morphodynamics with prior ischemic stroke in PV patients. In Table 2, we denote all patient characteristics along with laboratory data.

PV stroke patients were non-significantly younger (49 (45; 56) years) than patients without stroke (53.0 (41.5; 56.0) years). All patients with PV were stratified according to the NCCN thrombotic risk scale into ‘low’, ‘moderate’, and ‘high’ risk categories with only stroke patients in the high-risk group (*n* = 5), and only patients without stroke in the low-risk group (*n* = 17).

There was a statistically significant difference in aggregation amplitude (Amp) between patients with and without stroke (11.6 vs. 9.6 respectively, *p* = 0.03). Furthermore, RBC levels were significantly lower in PV stroke patients (4.8 vs. 6 respectively, *p* = 0.02).

We performed a logistic regression analysis (Table 3) to evaluate the possible associations of RBC deformability properties with ischemic stroke. The univariate analysis demonstrated only aggregation amplitude as marginally significantly associated with stroke in PV. In a model consisting of all but one (EImax) marker, there was a significant positive association between the time of formation of coin columns (Tf) (RBC rouleaux formation time constant) and the likelihood of ischemic stroke (OR 1.63 (95% CI = 1.06 to 2.52), *p* = 0.03). In addition, a higher aggregation index was associated with stroke (OR 1.17 (95% CI = 1.01 to 1.36), *p* = 0.04). The overall model was significant χ²(5) = 14.22, *p* = 0.01, Pseudo-R² (McFadden) = 0.27.

## 3. Discussion

Thrombotic complications, which are most common in patients with polycythemia vera compared with other myeloproliferative neoplasms, lead to disability and significantly contribute to mortality in the PV patient population [8]. Cerebral ischemia (stroke) can be the first presentation of PV. In a hospital clinical series, hematological pathology was observed in 1.27% (14/1099) of all consecutive in-hospital patients with first-ever stroke [18]. The highest incidence of thrombosis usually occurs shortly before or at the time of diagnosis and decreases over time, probably due to the effects of treatment. However, even with adequate treatment of PV and prophylaxis with acetylsalicylic acid, the risk of thrombosis remains. Significant risk factors include age (≥60 years) and a history of thrombosis. Changes in the number of blood cells (increase in the number of platelets, WBC, RBC) and high blood viscosity are also associated with an increased risk of thrombosis [19]. However, the study of the functional properties of cells in MPN was carried out only for platelets and WBC. Data on the functional state of RBC and the contribution of RBC to the development of thrombosis are extremely limited.

Red blood cell function (or dysfunction) is implicated in a wide range of pathologies and diseases. Polycythemia vera (PV) represents a model condition in that its defining feature is erythrocytosis. It is not unreasonable to expect differences in RBC morphodynamics between patients with and without PV. Thus, in the first step of our study, we aimed to identify possible biomarkers.

The lower maximal elongation index (EImax) was a hallmark of PV in our study. The deformability of RBCs is vital to their main function; even a slight decrease in RBC deformability causes a significant increase in microvascular flow resistance and blood viscosity, which may lead to thrombotic complications [20].

In contrast to EImax, the extent of aggregation (or amplitude [Amp]) was significantly higher in PV patients indicating possible increased vascular resistance, especially at the pre-capillary level [21]. Paradoxically, the smallest shear rate required for complete disaggregation (γ-dis or RBC disaggregation threshold) was statistically significantly lower in the setting of PV than in the control group. The latter finding may serve as evidence to compensatory reactions in PV patients which mitigate lower RBC deformability and impaired aggregation whilst maintaining adequate circulation.

RBC aggregation kinetics are described by the time constants Tf (which reflects the time course of 2D rouleaux formation) and Ts (which represents the formation of 3D structures resulting from secondary aggregation) [22]. In PV, a statistically significant negative correlation between both these time constants and γ-dis was observed—an association not found in the control group. A possible explanation may be that, in the setting of PV, there is an increase in adhesiveness of RBCs to the endothelium and longer Tf and Ts may serve to decrease blood effective viscosity [23].

Increased risk of thrombotic complications (including ischemic stroke) in PV patients is associated with high levels of hemoglobin, impaired rheology, and increased viscosity resulting from erythrocytosis, so the next step of our study included an analysis of the possible role of RBC morphodynamics in stroke occurrence in PV [24]. Apart from increased aggregation amplitude (Amp) in stroke patients, we did not observe any statistically significant differences between the two groups. This prompted us to do further uni- and multivariate logistic regression analysis, and we build a model consisting of five parameters of RBC deformability and aggregation which performed reasonably well (*p* < 0.01). An increase in amplitude (OR 1.40, 95% CI: 1.02–1.92), Tf (OR 1.63, 95% CI: 1.06–2.52), and AI (OR 1.17, 95% CI: 1.01–1.36) was associated with ischemic stroke. The aggregation index (AI) is a composite index that depends on both the extent and kinetics of aggregation. A higher AI was earlier found in patients with symptomatic carotid artery disease patients when compared to control [25]. The possible mechanisms of stroke occurrence in PV patients may act through effect upon blood viscosity and shear properties and are strongly influenced by biomechanical (RBC deformability) and surface (RBC aggregation) properties [26].

In this study, we highlighted the presence of changes in morphodynamics features of RBCs from patients with PV assessed using LoRRca. In this clinical setting, RBC alterations correlate well with cerebrovascular disease in the setting of PV, suggesting the existence of a possible link between erythrocyte morphodynamics and increased risk of stroke. In vivo studies of *JAK2 V617F* mice have demonstrated exacerbated vasoconstrictor responses resulting from increased endothelial oxidative stress caused by circulating RBC-derived microvesicles [27]. This may provide another possible link to thrombotic complications in patients with PV.

The primary goal of therapy in patients with PV is to reduce the risk of thrombosis by controlling hematocrit to <45%, a target associated with a reduction in CV death and major thrombotic events. The therapeutic tactics are based on determining the risk of thrombotic complications. Currently, for patients with PV, a prognostic index is used, developed in 2005, which includes variables such as age, a history of thrombosis, and concomitant diseases of the cardiovascular system. Patients at low risk (<60 years without a history of thrombosis) should be treated with phlebotomy and low-dose aspirin, while patients at high risk (≥60 years and/or with a history of thrombosis) should be given cytoreductive drugs.

Despite ongoing therapy (phlebotomy, acetylsalicylic acid, cytoreductive therapy), the risk of thrombosis remains high. It is known that RBCs with reduced deformability fail to pass the spleen, which acts as an RBC quality control organ [28]. Further research for risk factors will allow a more correct assessment of the likelihood of thrombotic complications and timely adjustment of ongoing therapy. Such a factor in patients with PV may be the morphodynamics of RBC.

The limitations of our study include a relatively small sample size, possible selection bias (since we included patients with PV from only one clinical center and not everyone agreed to continue the examination at the neurological center), and representation bias (not all patients diagnosed with PV during the study period did participate in this study).

## 4. Materials and Methods

### 4.1. Study Populations and Blood Collection

We enrolled in this cross-sectional study 48 patients with PV (according to WHO 2017 criteria). The control group consisted of 90 healthy subjects.

**Figure 4:**
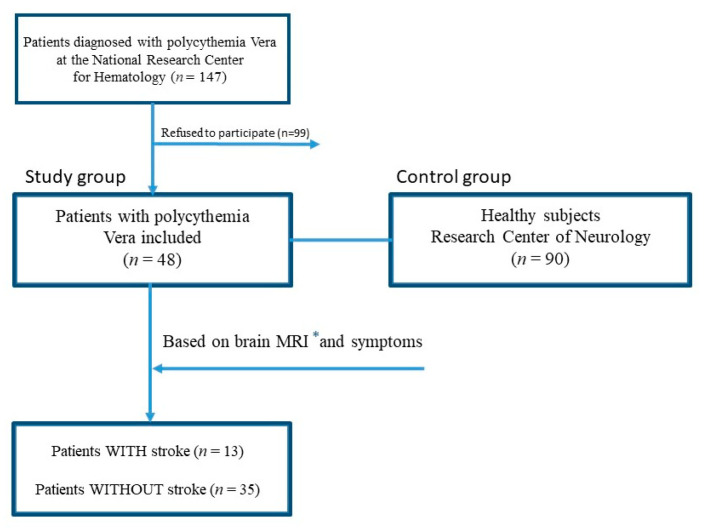
Flowchart study design. * *MRI—Magnetic resonance imaging*.

The study lasted from May 2015 to November 2021. In total, 147 patients were diagnosed with PV during this period (according to WHO 2017 criteria) at the National Research Center for Hematology (department of standardization and treatment methods), Moscow, Russia. Furthermore, 48 subjects who agreed to participate in the study were referred to the Research Center of Neurology, Moscow, Russia. All patients were further evaluated for the presence or absence of a stroke according to MRI and anamnesis (Figure 4).

All subjects underwent clinical examination, and complete blood count and morphodynamics features of RBCs were required for laboratory analyses. In all patients, a blood sample was obtained and collected into EDTA tubes. This study was carried out by the Declaration of Helsinki and approved by the local ethics research committee of the Research Center of Neurology. Written informed consent to participate was obtained from all subjects.

### 4.2. Measurement of Red Blood Cells Morphodynamics

The analysis of RBC morphodynamic features was performed using a laser-assisted optical rotational red cell analyzer (LoRRca MaxSis, Mechatronics, Hoorn, The Netherlands), according to the manufacturer’s instructions, as detailed below [29].

To obtain osmotic gradient-dependent RBC deformability, 50 μL of whole blood is suspended in 1 mL of polyvinylpyrrolidone buffer (Mechatronics, Hoorn, The Netherlands) and used for the analysis. The osmotic gradient curve generated by the instrument shows the variation in deformability as a continuous function of the osmolality of the solution in which RBCs are dissolved.

The method is based on the use of the Couette flow geometry with a fixed head and a rotating cylinder (glass) to create a simple shear flow. A thin layer of erythrocyte suspension is distributed between two concentric cylinders. The rotation of the outer cylinder (glass) causes deformation (elongation) of the RBC. With the help of a video camera, the diffraction indices of the laser beam, which fixes this deformation, are taken, followed by computer analysis of the data obtained. In whole blood, various indicators of erythrocyte aggregation are determined by measuring the reflected signal. A laser diode installed in the functional head is used as a light source; the reflected signal is detected by a photodiode. The LoRRca analyzer performs automatic measurement of RBCs, and the deformability of RBC is evaluated as an erythrocyte elongation index as a continuous function of the osmolar pressure of the suspension medium. Information about the shape and position of RBC relative to the osmolality axis in the osmoscanner graph makes it possible to judge the deformability of cells (at a given stress threshold), as well as the intracellular viscosity and the value of the surface volume of RBC. The erythrocyte index elongation program provides data on stress dependence (erythrocyte deformability curve) and dependence on exposure time (stability test). A syllectogram curve is generated at the end of the assay and the following parameters are automatically calculated: the aggregation amplitude (Amp, c.u.), showing the total extent of aggregation; the time of formation of coin columns (RBC rouleaux formation time constant) (Tf, s.); three-dimensional aggregates (Ts, s.); the aggregation index (AI, c.u.), calculated as integral of the total syllectogram curve; the rate of complete disaggregation (γ-dis, c.u.), i.e., a parameter that reflects the force required for the destruction of erythrocyte aggregates; and the deformability of RBC (EImax, c.u.). (Baskurt et al., 2009) [22].

### 4.3. Statistics

Statistical analysis was performed with R programming language (version 4.0.5) in an integrated environment (RStudio, version 1.3.1093, Boston, MA, USA); the following packages were used: “tidyverse”, “jtools”, “ggcorrplot”, “reshape2”, and “ggstatsplot”. The following non-parametric methods were used: the median and interquartile range for descriptive statistics, the Mann–Whitney U test when comparing two independent samples, the 2-sample test for equality of proportions with continuity correction, and Spearman correlation. Univariate and multivariable logistic regression was performed. Relative values of RBC morphodynamics parameters were visualized in a heatmap; the data transformation was performed via the ‘percentize’ function by using the empirical distribution function of the variables on their values, bringing each value to its empirical percentile (the percent of observations with that value or below it). Hierarchical clustering was performed by the ‘optimal leaf ordering’ algorithm; the resulting dendrograms are presented. All statistical tests were two-sided and were performed with an alpha level of 0.05. The null hypothesis was rejected if *p* < 0.05.

## 5. Conclusions

Thrombotic complications in MPN and PV, in particular, represent an important problem, as they are the main cause of disability and mortality in patients. The most important risk factors are advanced age and a history of thrombosis. The goal of therapy is to reduce the risk of thrombosis. Determination of the morphodynamics properties of RBC will allow a more correct assessment of the risk of thrombosis and timely adjustment of the treatment.

## Figures and Tables

**Table 1:** RBC morphodynamics in PV patients and control.

	PV (*n* = 48)	Control (*n* = 90)	*p*
EImax	0.47 (0.44; 0.51)	0.51 (0.47; 0.54)	0.0003539
Amp	10.1 (8.6; 12.2)	7.7 (6.6; 9.2)	0.000006
Tf, s	2.7 (1.9; 4.9)	3.5 (2.5; 5.4)	0.1784
Ts, s	19.8 (15.5; 33.3)	20.8 (16.2; 27.9)	0.4032
AI	53.9 (39.7; 64.9)	52.3 (43.2; 62.5)	0.9693
γ-dis, s^−1^	100 (100; 140)	140 (106; 188)	0.00009

**Table 2:** Main demographic and clinical characteristics in the study group.

	PV All (*n* = 48)	PV Stroke (*n* = 13)	PV No Stroke (*n* = 35)	*p*
EImax	0.47 (0.44; 0.51)	0.46 (0.38; 0.52)	0.47 (0.44; 0.51)	0.7186
Amp	10.1 (8.6; 12.2)	11.6 (10.0; 13.3)	9.6 (7.8; 11.4)	0.03573
Tf, s	2.7 (1.9; 4.9)	2.4 (1.9; 4.5)	2.7 (1.9; 5.0)	0.9445
Ts, s	19.8 (15.5; 33.3)	17.4 (15.6; 31.9)	19.9 (15.8; 33.9)	0.9076
AI	53.9 (39.7; 64.9)	53.9 (39.7; 63.6)	52.5 (39.7; 64.9)	0.9416
γ-dis, s^−1^	100 (100; 140)	100 (100; 100)	100 (100; 150)	0.6081
Age, years	51.5 (42.8; 56)	49 (45; 56)	53.0 (41.5; 56.0)	0.8891
*JAK2*, %	23 (13.5; 40.0)	15 (14;33)	24 (13;45)	0.4568
Ht, %	45 (42; 49.5)	43 (42; 46)	46 (42; 50)	0.5357
Hb (g/L)	157 (144;168)	152 (144;166)	158 (146; 169)	0.8254
RBC (×10^12^/L)	5.6 (4.9; 6.5)	4.8 (4.5; 5.7)	6 (5.2; 6.7)	0.02429
PLT (×10^9^/L)	373 (259; 526)	271 (228; 505)	374 (296; 527)	0.4864
Hydrea	34 (71%)	10 (77%)	24 (68.6%)	0.8349
ASA	43 (90%)	10 (77%)	33 (94%)	0.2231
NCCN risk				
low	17 (35.4%)	0 (0)	17 (48.6%)	0.005
moderate	26 (54.2%)	8 (61.5)	18 (51.4%)	0.7651
high	5 (10.4%)	5 (38.4%)	0 (0)	0.0008

**Table 3:** Univariate and multivariable logistic regression analysis of RBC deformability parameters and their association with ischemic stroke in PV.

	Univariate Analysis		Multivariable Analysis		Model Summary
	OR (95% CI)	*p*	OR (95% CI)	*p*	
EImax	0.04 (0–93.1)	0.41	Not included in the model		χ²(5) = 14.22 *p* = 0.01 Pseudo-R² (McFadden) = 0.27
Amp	1.28 (1.0–1.64)	0.05	1.40 (1.02–1.92)	0.04	
Tf	1.03 (0.87–1.21)	0.76	1.63 (1.06–2.52)	0.03	
AI	1.0 (0.96–1.04)	0.89	1.17 (1.01–1.36)	0.04	
Ts	1.0 (0.96–1.04)	0.99	1.04 (0.96–1.12)	0.37	
ydis	1.0 (0.99–1.00)	0.28	0.98 (0.96–1.00)	0.07	

## Data Availability

The data that support the findings of this study are available from the corresponding author, P.K., upon reasonable request.

## Abbreviations

PV	polycythemia vera
RBC	red blood cell
Amp	aggregation amplitude (extent of aggregation)
Tf	time of formation of coin columns (RBC-rouleaux formation time constant)
Ts	time of formation of three-dimensional aggregates (RBC-3D aggregate formation contribution)
AI	aggregation index
γ-dis	the rate of complete disaggregation
EImax	maximal elongation (deformability) index
WBC	white blood cell
MRI	Magnetic resonance imaging

## References

[1] Michiels J.J., Berneman Z., Schroyens W., De Raeve H. (2015). Changing concepts of diagnostic criteria of myeloproliferative disorders and the molecular etiology and classification of myeloproliferative neoplasms: From Dameshek 1950 to Vainchenker 2005 and beyond. Acta Haematol..

[2] Barbui T., Thiele J., Gisslinger H., Kvasnicka H.M., Vannucchi A.M., Guglielmelli P., Orazi A., Tefferi A. (2018). The 2016 WHO classification and diagnostic criteria for myeloproliferative neoplasms: Document summary and in-depth discussion. Blood Cancer J..

[3] Xia D., Hasserjian R.P. (2016). Molecular testing for JAK2, MPL, and CALR in myeloproliferative neoplasms. Am. J. Hematol..

[4] Seif F., Khoshmirsafa M., Aazami H., Mohsenzadegan M., Sedighi G., Bahar M. (2017). The role of JAK-STAT signaling pathway and its regulators in the fate of T helper cells. Cell Commun. Signal..

[5] Hultcrantz M., Björkholm M., Dickman P.W., Landgren O., Derolf Å.R., Kristinsson S.Y., Andersson T.M.L. (2018). Risk for arterial and venous thrombosis in patients with myeloproliferative neoplasms: A population-based cohort study. Ann. Intern. Med..

[6] Arboix A., Jiménez C., Massons J., Parra O., Besses C. (2016). Hematological disorders: A commonly unrecognized cause of acute stroke. Expert Rev. Hematol..

[7] Skoda R.C., Duek A., Grisouard J. (2015). Pathogenesis of myeloproliferative neoplasms. Exp. Hematol..

[8] Griesshammer M., Kiladjian J.-J., Besses C. (2019). Thromboembolic events in polycythemia vera. Ann. Hematol..

[9] Tefferi A., Elliott M. (2007). Thrombosis in myeloproliferative disorders: Prevalence, prognostic factors, and the role of leukocytes and JAK2V617F. Semin. Thromb. Hemost..

[10] Vannucchi A.M. (2009). Insights into the pathogenesis and management of thrombosis in polycythemia vera and essential thrombocythemia. Intern. Emerg. Med..

[11] Tefferi A., Pardanani A. (2015). Myeloproliferative neoplasms: A contemporary review. JAMA Oncol..

[12] Shallis R.M., Wang R., Davidoff A., Ma X., Podoltsev N.A., Zeidan A.M. (2020). Epidemiology of the classical myeloproliferative neoplasms: The four corners of an expansive and complex map. Blood Rev..

[13] Buks R., Dagher T., Rotordam M.G., Alonso D.M., Cochet S., Gautier E.-F., Chafey P., Cassinat B., Kiladjian J.-J., Becker N. (2021). Altered Ca^2+^ homeostasis in red blood cells of polycythemia vera patients following disturbed organelle sorting during terminal erythropoiesis. Cells.

[14] Man Y., Kucukal E., An R., Watson Q.D., Bosch J., Zimmerman P.A., Little J.A., Gurkan U.A. (2020). Microfluidic assessment of red blood cell mediated microvascular occlusion. Lab Chip.

[15] Porro B., Conte E., Zaninoni A., Bianchi P., Veglia F., Barbieri S., Fiorelli S., Eligini S., Di Minno A., Mushtaq S. (2021). Red blood cell morphodynamics: A new potential marker in high-risk patients. Front. Physiol..

[16] Byrnes J.R., Wolberg A.S. (2017). Red blood cells in thrombosis. Blood.

[17] Weisel J.W., Litvinov R.I. (2019). Red blood cells: The forgotten player in hemostasis and thrombosis. J. Thromb. Haemost..

[18] Arboix A., Besses C. (1997). Cerebrovascular disease as the initial clinical presentation of haematological disorders. Eur. Neurol..

[19] Finazzi G. (2004). A prospective analysis of thrombotic events in the European collaboration study on low-dose aspirin in polycythemia (ECLAP). Pathol. Biol..

[20] Kim J., Lee H., Shin S. (2015). Advances in the measurement of red blood cell deformability: A brief review. J. Cell. Biotechnol..

[21] Connes P., Lamarre Y., Waltz X., Ballas S.K., Lemonne N., Etienne-Julan M., Hue O., Hardy-Dessources M.-D., Romana M. (2014). Haemolysis and abnormal haemorheology in sickle cell anaemia. Br. J. Haematol..

[22] Baskurt O.K., Üyüklü M., Hardeman M.R., Meiselman H.J. (2009). Photometric measurements of red blood cell aggregation: Light transmission versus light reflectance. J. Biomed. Opt..

[23] Wautier M.-P., El Nemer W., Gane P., Rain J.-D., Cartron J.-P., Colin Y., Le Van Kim C., Wautier J.-L. (2007). Increased adhesion to endothelial cells of erythrocytes from patients with polycythemia vera is mediated by laminin α5 chain and Lu/BCAM. Blood.

[24] Wang J. (2003). Hyperviscosity in polycythemia vera and other red cell abnormalities. Semin. Thromb. Hemost..

[25] Mury P., Faes C., Millon A., Mura M., Renoux C., Skinner S., Nicaise V., Joly P., Della Schiava N., Lermusiaux P. (2017). Higher daily physical activity level is associated with lower RBC aggregation in carotid artery disease patients at high risk of stroke. Front. Physiol..

[26] Gillespie A.H., Doctor A. (2021). Red blood cell contribution to hemostasis. Front. Pediatr..

[27] Poisson J., Tanguy M., Davy H., Camara F., El Mdawar M.-B., Kheloufi M., Dagher T., Devue C., Lasselin J., Plessier A. (2020). Erythrocyte-derived microvesicles induce arterial spasms in JAK2V617F myeloproliferative neoplasm. J. Clin. Investig..

[28] Huisjes R., Bogdanova A., Van Solinge W.W., Schiffelers R.M., Kaestner L., Van Wijk R. (2018). Squeezing for life—Properties of red blood cell deformability. Front. Physiol..

[29] Zaninoni A., Fermo E., Vercellati C., Consonni D., Marcello A.P., Zanella A., Cortelezzi A., Barcellini W., Bianchi P., Zaninoni A. (2018). Use of Laser assisted optical rotational cell analyzer (LoRRca MaxSis) in the diagnosis of RBC membrane disorders, enzyme defects, and congenital dyserythropoietic anemias: A monocentric study on 202 patients. Front. Physiol..

